# The Intergenerational Transmission of Family Dissolution: How it Varies by Social Class Origin and Birth Cohort

**DOI:** 10.1007/s10680-023-09654-7

**Published:** 2023-02-23

**Authors:** Alessandro Di Nallo, Daniel Oesch

**Affiliations:** 1 Department of Social and Political Sciences, Bocconi University, Milano, Italy; 2 Swiss Centre of Expertise in Life Course Research LIVES & FORS, University of Lausanne, Lausanne, Switzerland

**Keywords:** Union dissolution, Social class, Intergenerational transmission of divorce, Family, Inequality

## Abstract

Children from separated parents are more likely to also experience the dissolution of their own union. For many children, parental separation thus is an adverse life course event that follows them into adulthood. We examine whether parents’ social class mitigates this adversity and weakens the intergenerational transmission of family dissolution for children from advantaged class origins. This is the case if separated parents with more resources are able to offer better living conditions to their children and keep them longer in education, reducing children’s incentives for early home-leaving, early cohabitation and early childbearing—three life course choices that increase the risk of later family dissolution. We analyse the existence of such a compensatory class advantage for three birth cohorts in the UK. Based on 38,000 life histories from two panel surveys (BHPS, UKLHS), we find a strong link between parents’ family dissolution and offspring’s family dissolution, and a reversal in the effect of parents’ class on children’s risk of family dissolution over the three birth cohorts of the Silent Generation (1925–45), Baby Boomers (1946–64) and Generation X (1965–79). However, there is no evidence that the intergenerational transmission of union dissolution is mitigated by a compensatory class effect for offspring from more advantaged class origins. Regardless of class origin, parents’ union dissolution is associated with a much larger risk of union dissolution among their offspring.

## Introduction

Children of separated parents tend to experience worse educational, health and well-being outcomes (Amato & Anthony, 2014; Härkönen et al., 2017; Leopold & Kalmijn, 2016). Moreover, the effect of parental separation extends to adulthood as children of separated parents are also more likely to witness the dissolution of their own couple. This phenomenon of intergenerational transmission of union dissolution has been observed in multi-country studies (Diekmann & Schmidheiny, 2013; Dronkers & Härkönen, 2008, Wagner & Weiss, 2006) as well as single-country analyses for Britain (Kiernan & Cherlin, 1999), Germany (Diekmann & Engelhardt, 1999), Italy (Todesco, 2013), the Netherlands (Liefbroer & Elzinga, 2012), Norway (Lyngstad & Engelhardt, 2009), Sweden (Gähler et al., 2009) or the USA (Amato & Patterson, 2017).

For many children, parental separation thus appears as a critical life event with often adverse effects that spill over into adulthood. The question we raise here is whether parents’ social class mitigates this effect and weakens the intergenerational transmission of separation for children from more advantaged socio-economic backgrounds. This is the case if separated parents with more resources are able to offer better living conditions to their children and keep them longer in education, reducing children’s incentives for early home-leaving, early cohabitation and early childbearing—three life course choices that increase the risk of later family dissolution (Gähler et al., 2009; Kuperberg, 2014).

In general, research in social stratification suggests that children from advantaged social backgrounds are less hampered in their educational and occupational trajectories by unfavourable life events (Bernardi, 2014; Bernardi & Grätz, 2015; Bernardi & Gil-Hernandez, 2020). However, it is unclear whether such a compensatory advantage linked to social class also mitigates the consequences of parental separation. While some studies suggest that it does so in terms of schooling (Albertini & Dronkers, 2009; Grätz, 2015), other studies find a more detrimental effect of parental divorce on education for children from higher than lower-class backgrounds (Bernardi & Boertien, 2016, 2017a; Bernardi & Radl, 2014; Martin, 2012).

A growing body of research examines how parental separation affects children’s education depending on their social origin (see the review by Bernardi & Boertien, 2017b). However, no study has so far examined whether the intergenerational transmission of union dissolution varies by parents’ social class. Our article’s primary contribution therefore is to investigate how parental class influences the intergenerational transmission of union dissolution. If parental break-up represents a larger misfortune in the lives of children from less advantaged origins that carries over into their own unions, this represents a major challenge for social policy.

Besides providing the first study on this question, our article wishes to make two additional contributions. First, we heed the advice that research on the intergenerational transmission of divorce should move beyond divorce and look at family instability more generally (Amato & Patterson, 2017). Our analysis thus focuses on the disruption of childbearing unions, regardless whether these unions are marital or cohabiting. This definition has the advantage of providing us with the same measure of separation for parents’ and children’s generation: the dissolution of a childbearing union. This focus also allows us to analyse those union dissolutions that have more far-reaching social consequences because they involve children (Cherlin, 2009). Moreover, it acknowledges that the risk of separation differs between couples with and without children (Kalmijn & Leopold, 2021).

Second, our study focuses on the UK and uses two leading panel datasets, the British Household Panel Survey 1991–2008 and Understanding Society 2009–2019. These panel data provide us with the life histories of 38,000 adults in the child generation who were born over the 20th century. This makes it possible to examine how the class pattern of the intergenerational transmission of family dissolution evolved over successive birth cohorts. Given the reversal in the educational gradient of divorce over the 20th century—higher education being no longer associated with higher, but lower risk of separation (Härkönen & Dronkers, 2006; Kalmijn & Leopold, 2021; Matysiak et al., 2014), parental separation and social class origin are likely to interact differently for children’s separation risks in younger than older cohorts.

Our article first discusses the mechanisms behind the intergenerational transmission of union dissolution and then elaborates why this transmission may vary by parental class and birth cohort. It then presents our data and measures of family dissolution and social class. The results show a strong link between parents’ family dissolution and offspring’s family dissolution, and a reversal in the effect of parents’ class on children’s risk of family dissolution over birth cohorts. However, there is no evidence that the intergenerational transmission of separation is mitigated by a compensatory class effect for offspring from more advantaged class origins.

## Theoretical Background

### Explaining the Intergenerational Transmission of Divorce

Parental separation is one of the best documented risk factors for union dissolution (Amato & DeBoer, 2001). A meta-analysis finds systematically higher risks of divorce for children of divorced parents in 19 Western countries studied (Wagner & Weiss, 2006, p. 491), a result confirmed by two comparative studies that analyse over a dozen countries each (Diekmann & Schmidheiny, 2013; Dronkers & Härkönen, 2008). The association between parental divorce and children’s divorce is strong. In a British cohort born in 1958, 44% of men from divorced families, but only 26% of men from intact families had witnessed the break-up of their own first partnership (Kiernan & Cherlin, 1999, p. 40). In France, 24% of the children of divorced parents had also divorced as compared to only 13% of children with non-divorced parents (Traag et al., 2000, p. 6).

Why does parents’ separation cast such a long shadow on their children’s future partnerships? Schematically, four mechanisms may contribute to the intergenerational transmission of union dissolution: genes, socialization, socio-economic resources and life course choices (Liefbroer & Elzinga, 2012; Moen et al., 1997).

Genetic inheritance likely matters for union dissolution if parents and their children share genetic traits that increase the risk of partnership problems such as neuroticism or depression. Evidence from twin studies in the USA (McGue & Lykken, 1992) and Australia (D’Onofrio et al., 2007) as well as a Swedish register study using an adoption design (Salvatore et al., 2018) all suggest that the intergenerational transmission of marital instability is not solely driven by environmental factors, but also due to genetic inheritance.

Subsumed under socialization processes, a second theoretical mechanism stresses the importance of social learning, emotional stability and parents as role models (Amato, 2000; Moen et al., 1997). Children develop interpersonal skills and values from observing parental models. They notably learn from divorced parents that dissatisfying marriages can be voluntarily ended (Amato & DeBoer, 2001). Besides creating stress, growing up in a divorced family may thus deprive children of role models for relationship skills and reduce their marital commitment (Amato & Patterson, 2017).

A third mechanism puts the focus on socio-economic resources (Liefbroer & Elzinga, 2012; Moen et al., 1997). Family disruption reduces the amount of resources that parents can pass on to their offspring, and children typically experience a drop in their standard of living after parental divorce (Aassve et al., 2007). As divorced families have less time and money to invest in their children’s education, these children are more likely to miss out on higher education (Bernardi & Radl, 2014; Kreidl et al., 2017). They receive less economic support from their parents when forming their own households and, at adult age, possess less wealth than peers from intact families (Bernardi et al., 2019; Lersch & Baxter, 2021). Less secure incomes increase economic stress and reduce union stability (Conger et al., 2010).

A fourth mechanism highlights life course transitions—transitions that may, in turn, be the consequence of socialization and economic insecurity. Growing up in a divorced household makes young adults more likely to leave the parental home early, to enter cohabitation early and to become parents early. They thus follow a pattern of early and often unstable demographic transitions that are associated with higher risks of family dissolution (Amato, 2010; Gähler et al., 2009; Kuperberg, 2014; Lyngstad & Jalovaara, 2010).

Empirical evidence is stronger for socialization, notably the lack of partnership commitment (Amato & DeBoer, 2001), and life course transitions (Gähler et al., 2009) as determinants of the intergenerational transmission of divorce than socio-economic resources (Wolfinger, 2005). Yet the pathways from parents’ to offspring’s union dissolution are diverse and likely involve a combination of genetic, socio-demographic and behavioural factors. Our aim is not to disentangle these pathways and to provide an unambiguous explanation of the transmission of union dissolution, but rather to determine the extent to which this transmission varies by parental social class.

### Heterogeneous Child Outcomes After Parental Separation

Earlier studies have analysed how parents’ union dissolution affects various child outcomes depending on parental social class. While none of these studies include offspring’s own separation as an outcome, they provide a theoretical perspective on heterogeneous effects that may usefully apply to the intergenerational transmission of union dissolution.

The key mechanism stems from research on educational inequality and is known as a compensatory class advantage (Bernardi, 2014). It stipulates that a drawback in early life likely persists or grows over time for children from lower-class parents, whereas higher-class parents have the resources to attenuate its effect for their children. It is in the moments of adversity that social origin kicks in and class differences between families become salient.

Parental separation may constitute an adverse life course event that tends to decrease the financial means and parental time available for children’s development. Parents in subordinate class positions, having fewer resources to begin with, may struggle more to limit the adverse financial consequences of their separation. This is the case if they have to move to smaller flats and cheaper neighbourhoods with lower-quality schools—or if they can no longer afford their children’s extra tuition. Non-material resources may also decline, notably parental involvement in children’s lives, and this decline may again be stronger for children from less advantaged backgrounds. Evidence from Germany (Grätz, 2017) and the Netherlands (Kalmijn, 2015) suggests that after parental separation, low-educated fathers decrease their involvement in children’s lives to a larger extent than high-educated fathers.

In this view, a parental break-up represents a larger misfortune in the lives of children from less advantaged origins. If their separated parents have fewer means to pay for education, provide less attractive housing and become less involved in their children’s lives, these children are more likely to quit education early, leave home early, cohabit early and bear children early. These four life course choices are fostered by parents’ union dissolution (Cherlin et al., 1995; Dahlberg, 2015; Gähler et al., 2009; Wiik, 2009) and may, in turn, increase offspring’s risk of union dissolution (Kuperberg, 2014).

With respect to educational outcomes, the empirical evidence is inconclusive as to whether upper-class children enjoy a compensatory class advantage after parental separation. Several studies suggest that the negative effect of parental separation is concentrated among children from less educated fathers in Germany (Grätz, 2015) and less educated mothers in Italy (Albertini & Dronkers, 2009), the Netherlands (Mandemakers & Kalmijn, 2014) and USA (Augustine, 2014). Yet other studies find the opposite result, namely that parental separation harms children’s education more if parents—and notably fathers—are highly educated in Britain (Bernardi & Boertien, 2016) and the USA (Martin, 2012) as well as in a host of European countries with educational systems that do not track students early (Bernardi & Radl, 2014). These contradictory results raise the prospect that parental separation may not systematically increase the risk of adverse outcomes for children from different classes.

### Cohort Changes in the Class Gradient of Divorce

The key variables of our study—union dissolution and social class—have been anything but stable over the life time of respondents in our study, roughly the last hundred years. The Second Demographic Transition has led to greater instability of married unions and fostered strong growth in the even less stable cohabiting unions (Kalmijn & Leopold, 2021). This “separation surge” has been increasingly concentrated among less educated people and the working class—who, in the wake of educational expansion and occupational upgrading, have become a smaller and possibly more negatively selected group of the society (Breen & Müller, 2020).

For these reasons, it is likely that the link between social origin and post-separation outcomes varies across cohorts. This argument is notably suggested by the reversal in the educational gradient of divorce. While the higher-educated were more likely to divorce over much of the 20th century, in the 21st century the lower-educated have higher separation rates in a growing number of Western countries (Härkönen & Dronkers, 2006; Kalmijn & Leopold, 2021; Matysiak et al., 2014; Musick & Michelmore, 2018). As long as divorce was a rare and stigmatized event that required legal and economic resources as well as resistance against dominant norms, members of the higher classes were more likely to separate (Goode, 1962; Härkönen & Dronkers, 2006). Yet once the liberal attitudes towards divorce began to trickle down the social hierarchy and divorce became more common, life strains such as financial needs and social isolation began to matter more for couples’ stability (Hogendoorn et al., 2021).

In analogy to the reversal of the educational gradient, the association between parents’ education and offspring’s risk of union dissolution may also have changed over time. Given that parents’ and children’s educational attainment continues to be strongly correlated in Europe and North America (Bernardi & Ballarino, 2016; Pfeffer, 2008), we expect the same association to hold between parents’ education and children’s risk of union dissolution as between children’s own education and own risk of union dissolution.

Available evidence indeed suggests that this reversal is underway. While several country studies find that higher parental education increases the separation risks among offspring in Finland (Mäenpää & Jalovaara, 2014), Italy (Todesco, 2013), Norway (Lyngstad, 2006) or Sweden (Gähler et al., 2009), a multi-country study shows that this relationship is reversing over time as having higher-educated parents is progressively associated with a *lower* risk of offspring’s family dissolution among younger cohorts in Europe, most notably so in the UK (Brons & Härkönen, 2018).

This finding suggests that the social stratification underlying the intergenerational transmission of union dissolution has changed over time and thus points to the possibility of variation across birth cohorts—variation that may stem from a host of factors such as shifts in social norms, costs linked to separation or selection. Regardless of the underlying mechanism, we expect to see a shift in the effect of parental class on offspring’s family stability over the successive birth cohorts of the 20th century. In analogy to the reversal of the educational gradient of divorce, we expect more advantaged parental class positions to have become gradually associated with lower rates of union dissolution among their offspring.

Crucially, we expect the compensatory class effect to reinforce this reversal by further reducing the risk of union dissolution for offspring from separated (upper-)middle class families over successive birth cohorts. Our hypothesis is that among younger cohorts, offspring from advantaged classes will be less affected by parents’ separation than offspring from disadvantaged classes. In other words, for children from advantaged backgrounds born after World War II, we expect a compensatory class advantage to set in after parental separation and thus to reduce the intergenerational transmission of union dissolution.

## Data, Measures and Method

### Data and Analytical Sample

Our analysis is based on longitudinal data from the British Household Panel Study (BHPS) 1991–2008 and its successor survey, Understanding Society (UKHLS) 2009–2019. We combine the two datasets in order to increase the number of observations and to cover longer periods in the life histories of different cohorts. Our focus is on the dissolution of childbearing unions and we therefore include in both the parental and child generation only individuals with children. Our focus is on the child generation and we limit our sample to individuals in the child generation born between 1925 and 1979. The observation window starts when a respondent in the child generation becomes a parent, and it ends with his or her separation, death, panel non-response, right-censoring after 2019 or after 30 years of a child-bearing union. The initial sample size is 46,196 individuals and 1,000,881 person-year observations. After excluding respondents with missing information on relationship histories or parents’ socio-economic status, we obtain an analytical sample of 38,515 individuals and 958,240 person-year observations from the child generation.

### Dependent Variable

Our dependent variable is family dissolution and measured as the break-up of one’s childbearing union, regardless of whether it was marital or cohabiting. By focusing on childbearing unions, we use the same definition of family dissolution for parents’ and children’s unions. This sets us apart from other studies that move beyond divorce, but use different measures for the two generations: divorce in parents’ generation, but dissolution of first partnership in offspring’s generation (Kiernan & Cherlin, 1999); family dissolution for parents’ generation, but separation of any co-resident cohabitation—with or without children—for offspring’s generation (Amato & Patterson, 2017).

Besides consistency, the focus on family unions has the additional advantage of social relevance as it puts the spotlight on those separations that have potentially negative implications for third parties, namely children (Cherlin, 2009). In this context, Kalmijn and Leopold (2021) remind us that only about half of all separations in Western Europe involve children and that the separation surge in the late 20th century was much stronger among couples without than among couples with children. Of the 38,515 respondents in our analytical sample, 6927 or 18% experienced the dissolution of their own childbearing union during our observation window.

### Independent Variables

Our first key independent variable is parents’ union dissolution. The two surveys ask whether respondents lived in the same household with both parents until the age of 16, allowing us to distinguish three groups: (a) respondents from intact families who lived with both parents until the age of 16; (b) respondents from non-intact families where one of the two parents moved out before the child was 16; (c) respondents from families where at least one parent died during the respondent’s childhood. In our analytical sample, 83% of individuals come from intact families, 9.4% from non-intact families and 7.6% from families where at least one parent died when the respondent was a child.

Our second key independent variable is parents’ social class. The divorce literature mostly uses education as a measure of individuals’ position in social stratification. Yet we would argue that social class based on individuals’ position within the labour market—their occupation—is a stronger determinant of the opportunities and constraints that people face in terms of life chances. Notably for the purpose of our study, it appears as a better proxy for the economic, social and cultural resources that parents can harness for their children. However, given the close link between educational and occupational attainment, the same mechanisms should hold for the two indicators, and we provide results with education as a robustness check.^1^

For parental social class, we use a merged version of the scheme developed by Oesch (2006) and distinguish four categories: (1) Upper-middle class, including professionals and managers; (2) Lower-middle class, including technicians, associate professionals and office clerks; (3) Skilled working class, including skilled sales and service workers as well as craft workers; (4) Low-skilled working class, including assemblers, machine operatives and elementary occupations in agriculture, production, construction, cleaning, sales and services.^2^ We use the dominance approach and attribute to each individual the higher class of either father or mother (Erikson, 1984). In terms of social origin, 25% of our analytical sample come from families of the upper-middle class, 26% from the lower-middle class, 22% from the skilled working class and 20% from the low-skilled working class, with missing information for 8% of respondents.

For all our analyses, we use a second stratification variable that is based on socio-economic status and measured with ISEI (International Socio-Economic Index of Occupational Status). This indicator reflects the mean earnings and education in a given occupation (Ganzeboom & Treiman, 1996) and has the double advantage of providing us with a linear measure and allowing us to attribute to each respondent the mean socio-economic status of *both parents*. Moreover, we use the normalized z-score of socio-economic status which standardizes the variable’s distribution by setting the mean at 0 and dividing values by the standard deviation. The normalized distribution of ISEI z-scores also allows us to run a robustness check that accounts for differential selection into low socio-economic positions over time when comparing different birth cohorts. By normalizing the distribution of parents’ socio-economic status within each birth cohort, we account for the massive upgrading of the British class structure over the 20th century (Bukodi & Goldthorpe, 2018).

Respondents were asked about their parents’ occupation when they were children and aged 14. Separation may thus have taken place before parental occupation was measured. However, this is unlikely to bias the analysis because people’s class positions are quite stable over time: The range of occupational options tends to be strongly constrained by initial educational attainment and job mobility is typically of a short-range nature between occupations set at a similar hierarchical level rather than across social classes (Mayer, 2000; Murphy, 2014).

Our third key independent variable is birth cohort and we distinguish three sociologically meaningful birth cohorts that capture similar historical contexts (Howe & Strauss, 1992): the Silent Generation 1925–1945, the Baby Boomers 1946–1964, and Generation X 1965–1979. In our analytical sample, 7999 respondents belong to the Silent Generation, 15,768 to the Baby Boomers, and 14,224 to Generation X. We show robustness tests for two alternative cohort variables: birth cohorts measured in decades and union cohorts.

All our models include three control variables: year of birth, gender and self-identified ethnicity (measured in 9 categories). Further variables that possibly mediate the effect of parental separation on children’s separation include children’s age at union formation (in years), partnership status (cohabiting or married) and education (degree; other higher education; A level or similar; GCSE or similar; other qualification; no qualification or missing). Table 3 in the appendix provides descriptive statistics for all variables used in the analysis.

### Model

Our model estimates the effects of parental class, parental family dissolution and the interaction between these two variables on offspring’s family dissolution. The model is shown in the following equation:

$$Y_{jt} = \beta {\text{Class}}_{j} + \delta {\text{Parent}}\_{\text{Diss}}_{j} + \gamma {\text{Class}}_{j} *{\text{Parent}}\_{\text{Diss}}_{j} + \zeta {\text{Controls}}_{jt} + \phi \left( t \right) + \upsilon_{jt}$$

where $Y_{jt}$ is a binary measure of family dissolution for respondents *j* in the child generation at time *t*. ${\text{Class}}_{j}$ indicates parents’ social class, either operationalized as a categorical class measure or a continuous ISEI-score, and $\beta$ represents the associated coefficients. ${\text{Parent}}\_{\text{Diss}}_{j}$ indicates whether individuals experienced their parents’ union dissolution before the age of 16, and this variable of parental dissolution is interacted with parental class. The coefficient associated with the interaction term, $\gamma ,$ captures the differential effect of parental separation on individuals’ childbearing unions by parental social class. ${\text{Controls}}_{j}$ stand for socio-demographic control variables such as year of birth, age, gender, and ethnicity. Finally, $\phi \left( t \right)$ captures the duration of the union in years.

We estimate discrete-time event history regressions after transforming our data into discrete-time event history format, with person-years as the unit of analysis. This provides us with predicted probabilities of family dissolution that vary between 0.5 and 1.5% per year in the full sample. These event history analyses also enable us to account for attrition and, hence, include individuals who reported their family histories, but dropped out before the last survey wave. Therefore, we do not impose any minimal age threshold as a sample restriction on this event history model. Another advantage is to account for the influence of union duration. The time function is modelled with a linear, quadratic and cubic term of years since the union start.

We validate all our results with linear probability models (LPM) which have two attractive features. They allow us to directly compare the coefficients of different models (Mood, 2010), and they provide us with an intuitive metric, namely the cumulative predicted probability of family dissolution. As linear probability models do not account for right-censoring (the fact that the outcome has not yet occurred for everyone by the time of the interview), we specifically limit the analytical sample for these models to respondents who were at least 40 years old and had thus some time for family formation and separation. This leads to a slightly smaller sample (*N* = 34,027). In this analytical sample, the proportion of respondents who had experienced the dissolution of their child-bearing union was 16% among the offspring of intact families and 29% among those of non-intact families.

## Results

### Intergenerational Transmission of Family Dissolution by Parental Class

**Fig. 1:**
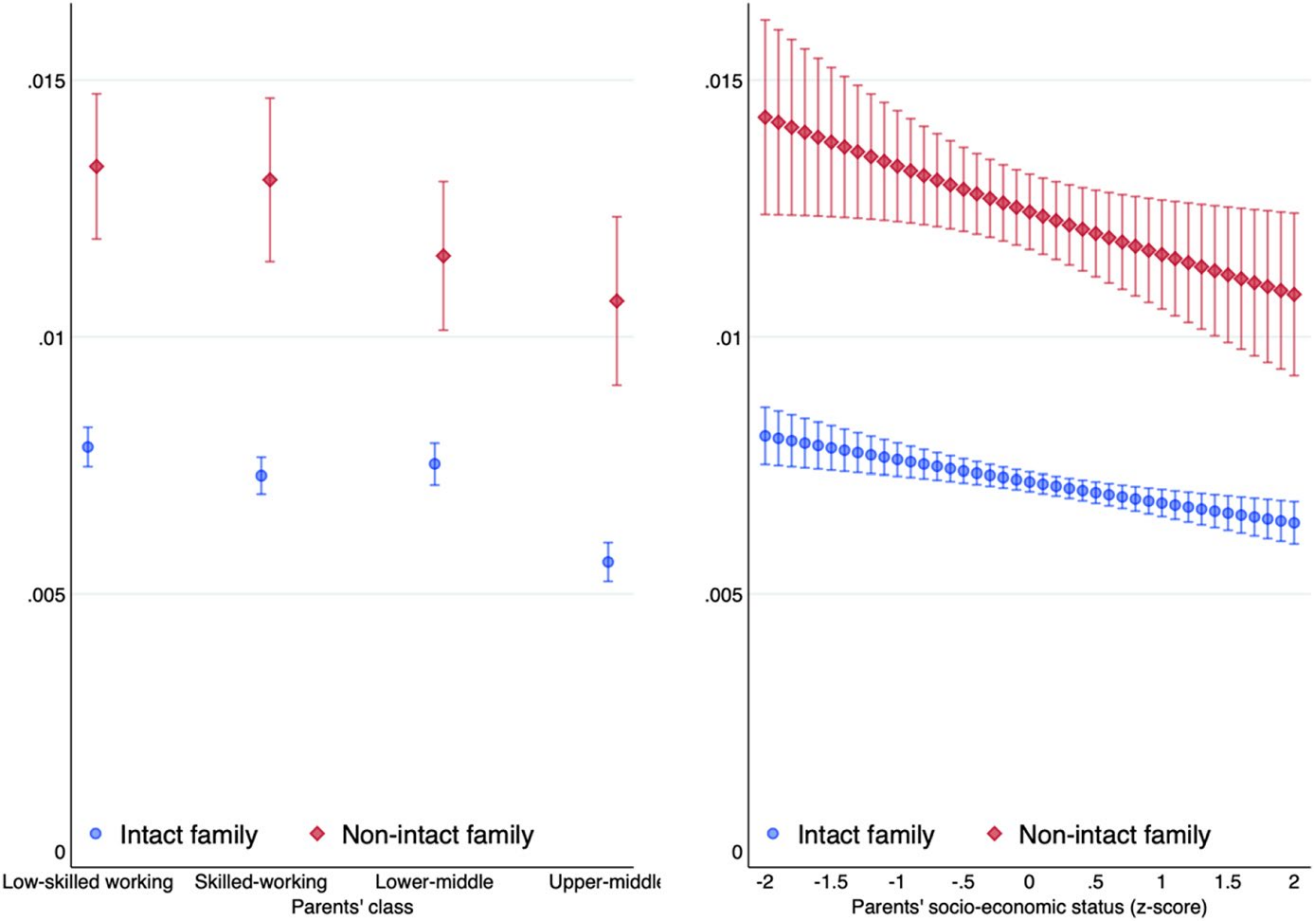
Predicted annual probability of offspring’s family dissolution by parents’ social class (right) and socio-economic status (left). Discrete-time event history model

We first compare in Fig. 1 the predicted probability that offspring from intact and non-intact families experience the dissolution of their own childbearing union, depending on their social origin. The left-hand panel measures social origin with parents’ social class and the right-hand panel with parents’ socio-economic status (ISEI). Figure 1 shows the annual probability of family dissolution based on event history analysis, whereas the cumulative probability based on LPM is shown in Fig. 5 in the appendix. All these models control for year of birth, gender, ethnicity and union duration.

Individuals who experienced their parents’ separation when growing up are much more likely to also see their own childbearing relationship break-up than individuals who grew up in intact families. On an annual basis, the separation rate is about 1.2% for offspring from non-intact families as compared to 0.7% for offspring from intact families. Regardless of whether social origin is measured with class or socio-economic status, we observe a negative socio-economic gradient of family dissolution. This means that children from upper-middle class families face a lower risk of seeing their couple break-up than children from working-class parents. This socio-economic gradient looks very similar for class and socio-economic status—and thus suggests that the observed relationship is not due to the idiosyncrasy of a given social stratification indicator. Indeed, the negative gradient is also visible if parents’ social position is measured with education. The children of parents who hold a tertiary degree are less likely to separate from their partners than the children of parents who left school without any qualification (see Fig. 6 in the appendix).

Crucially, these results provide no evidence for a heterogeneous effect of class on the intergenerational transmission of family dissolution. The negative class gradient of family dissolution looks very similar for children from intact and non-intact families. Offspring from intact families enjoy greater union stability than offspring from non-intact families, regardless of parents’ social class or socio-economic status. At first sight, we therefore observe no compensatory class effect for upper-middle class children who grew up in non-intact families.

Rather than to solely rely on graphical interpretation, we show in Table 1 the odds ratios of the event history analysis. These results confirm that parents’ family dissolution is a major risk factor for the stability of offspring’s own couples. Children experiencing their parents’ separation have twice the odds to separate themselves. In terms of cumulative probabilities as shown with the LPM, this means that their likelihood of family dissolution is 12 percentage points higher than for children who grew up with both parents (see Table 4 in the appendix). With respect to social origin, the main contrast is between offspring from the upper-middle and low-skilled working class. Offspring from upper-middle class parents have odds to separate that are 35% lower than offspring from the low-skilled working class.

**Table 1:** The effect of parents’ family status and social class on offspring’s family dissolution. Discrete-time event history model

	Odds	SE	Odds ratio	SE
*Parents’ family status (ref: intact family)*				
Non-intact family	2.09***	(0.104)	1.71***	(0.103)
*Parents’ social class (ref: low-skilled working class)*				
Skilled working class	0.91**	(0.039)	0.93**	(0.033)
Lower-middle class	0.91**	(0.041)	0.96	(0.036)
Upper-middle class	0.65***	(0.034)	0.72***	(0.031)
*Parents’ family status # parents’ social class*				
Non-intact # skilled working class			1.06	(0.096)
Non-intact # lower-middle class			0.91	(0.083)
Non-intact # upper-middle class			1.12	(0.118)
Observations (individuals)	38,515		38,515	

Controls are included for year of birth, gender and ethnicity

*p < .05

**p < .01

***p < .001

While the main effects of parents’ family status and parents’ social class are sizeable and statistically significant, the interaction effects between these two variables are tiny and not statistically significant. Contrary to our expectation, the intergenerational transmission of union dissolution does not interact with parental class.

### Mediating Variables Between Parents’ and Offspring’s Separation

To what extent is the intergenerational transmission of family dissolution mediated by life course transitions or by resources such as one’s own education? We try to answer this question by estimating four nested models (see Table 5 in the appendix). A first model only includes the two main variables of parental family status and parental class as well as the interaction between these two variables. A second model adds three socio-demographic controls: year of birth, gender and ethnicity—this is our preferred model that we used for Fig. 1. A third model adds three measures of life course transitions: type of union (married or cohabiting), age at union formation, and being married in the past. A fourth and final model further includes a respondents’ own education.

**Fig. 2:**
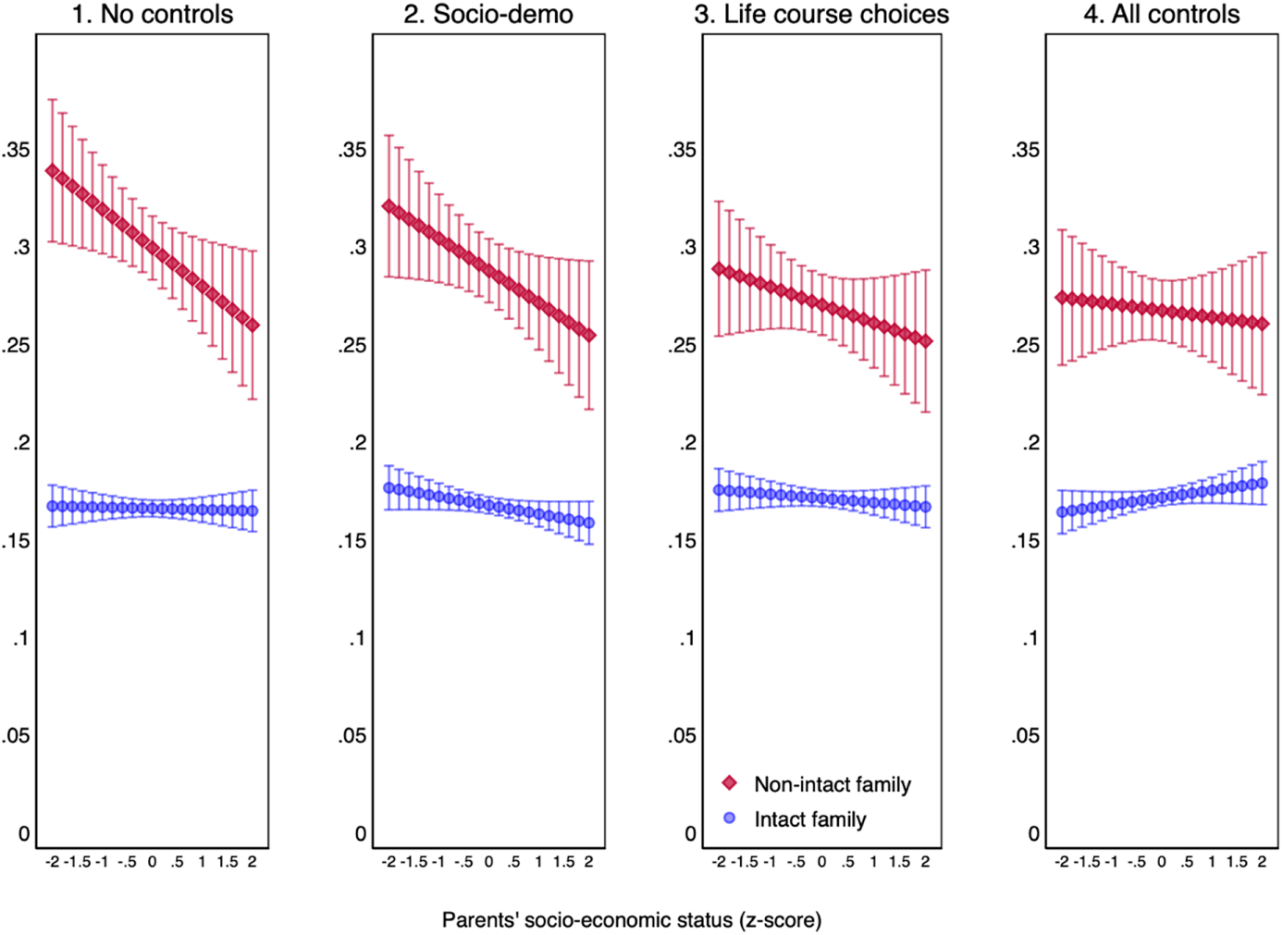
Cumulative predicted probability of offspring’s family dissolution by parents’ family status and socio-economic status, controlling for life course transitions (in %). Linear probability model

What are the findings? Figure 2 shows that throwing the kitchen sink of life course transitions at our regression has only a marginal influence on the intergenerational transmission of separation. Having a younger age at family formation, cohabiting, having had other unions with children before and quitting education early are all associated with a higher risk of family dissolution. However, these transitions do not mediate the effect of parents’ unstable union on offspring’s unstable union: the gap in the dissolution rate between offspring from intact and non-intact unions remains unchanged. Crucially, our conclusion remains unchanged that there is no interaction between parents’ family status and parents’ class (see Table 5in the appendix).

Note also that the introduction of variables related to socio-demographics, union formation or education does not contribute to closing the large family-dissolution gap between offspring from intact and non-intact families. However, once we introduce measures on life course choices and own education, a more advantaged paternal background is no longer associated with a lower risk of family dissolution. Children from less advantaged families seem more likely to experience the dissolution of their couple because they tend to be younger when entering a child-bearing union, to cohabit rather than to marry and to leave the education system with lower qualifications. A more disadvantaged class origin therefore leads to life course transitions that are associated with more union instability.

### Differences Across Birth Cohorts

Given the spectacular reversal in the educational gradient of divorce over the last decades (Härkönen & Dronkers, 2006), we expect to see differences by birth cohorts. We thus examine how the intergenerational transmission of family dissolution varies by social class for the three cohorts of Silent Generation, Baby Boomers and Generation X.

**Fig. 3:**
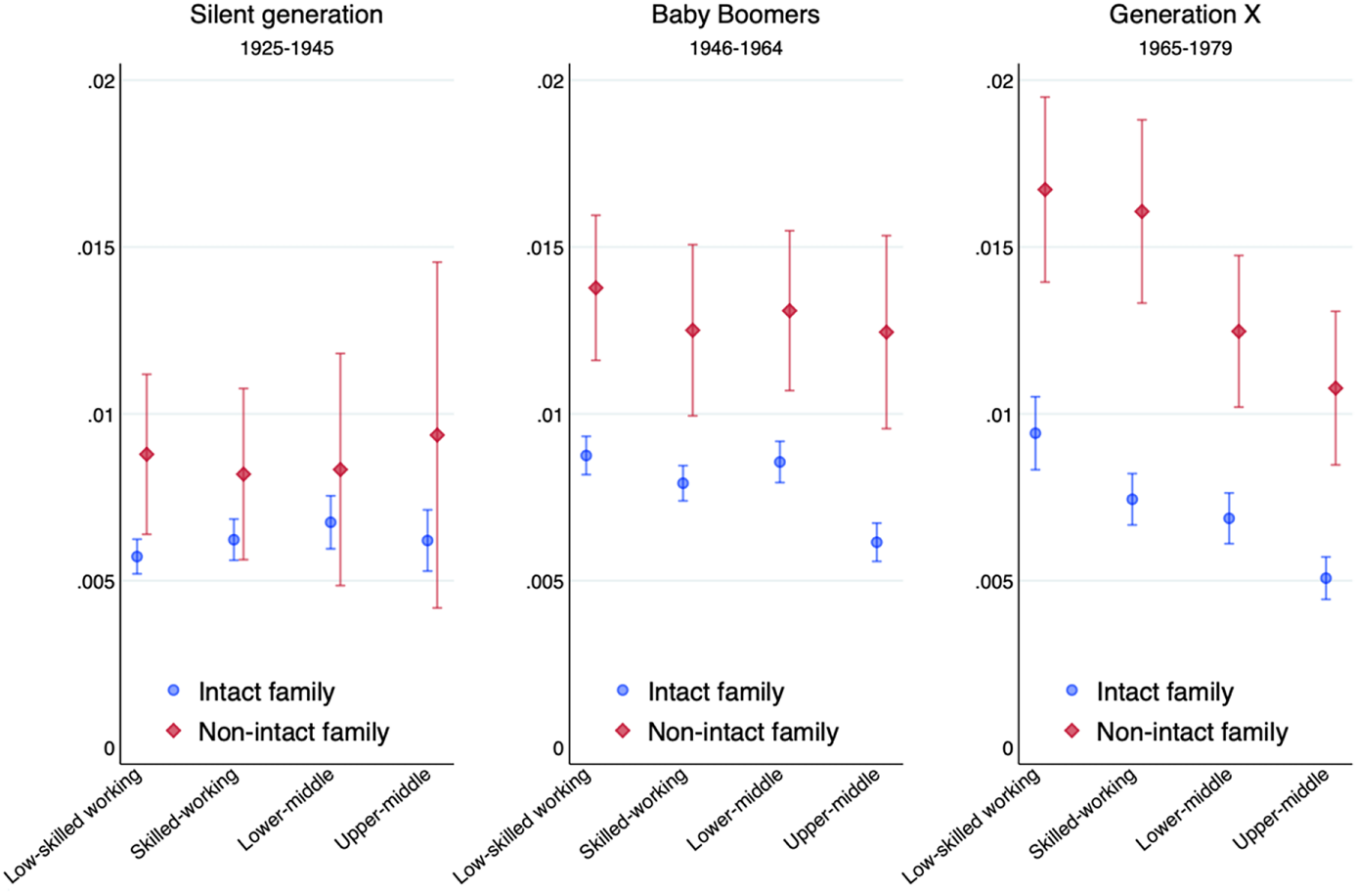
Predicted annual probability of offspring’s family dissolution by parents’ class for three birth cohorts. Discrete-time event history model

**Fig. 4:**
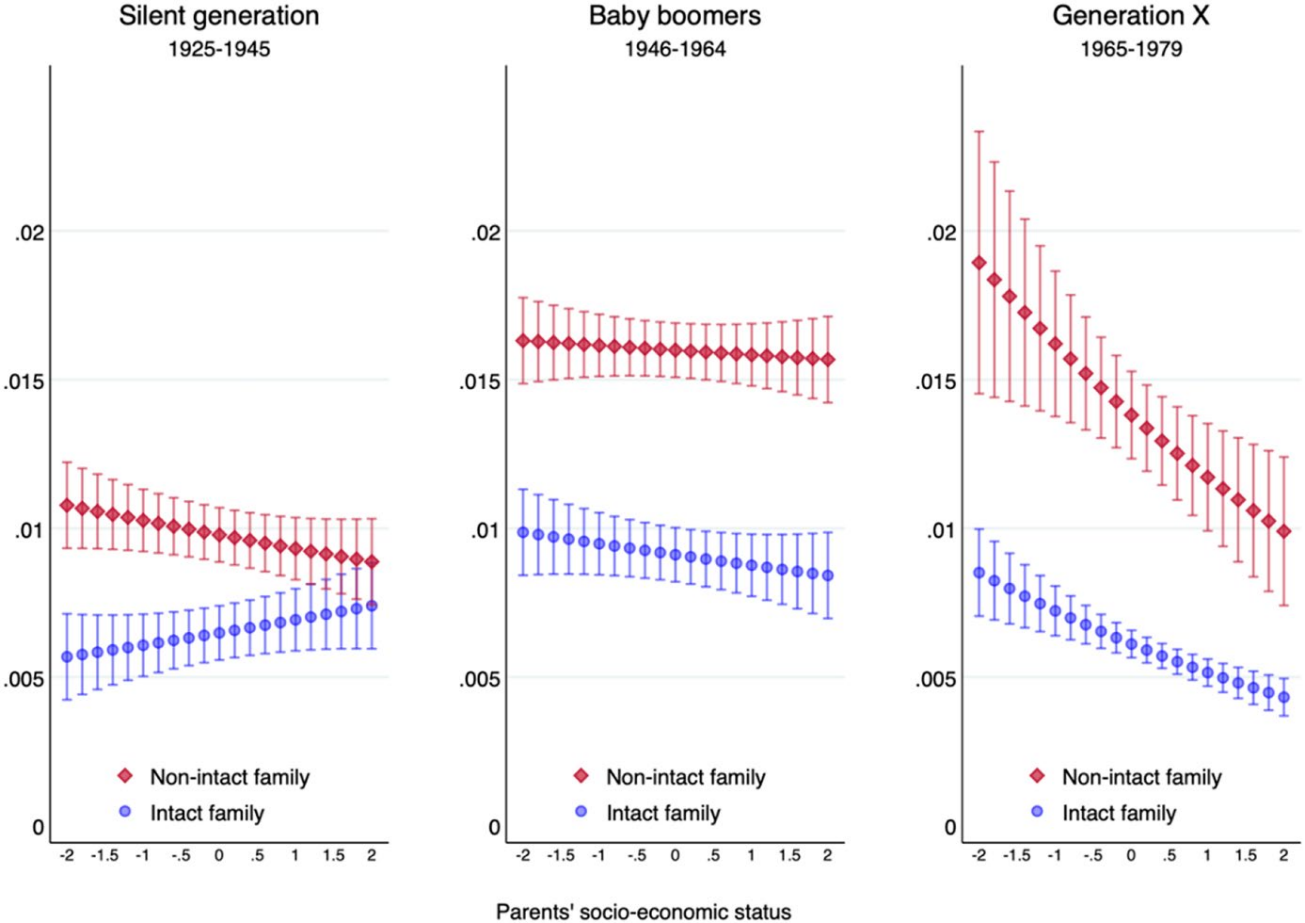
Predicted annual probability of offspring’s union dissolution by parents’ socio-economic status for three birth cohort. Discrete-time event history model

**Fig. 5:**
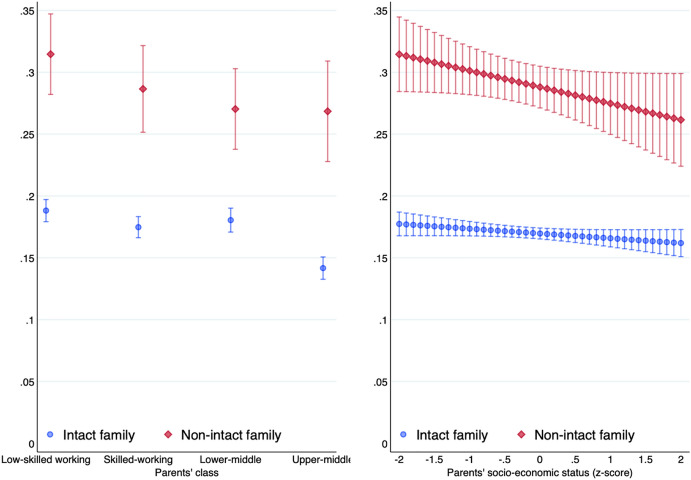
Cumulative predicted probability of offspring’s family dissolution by parents’ class (right) and socio-economic status (ISEI, left), in %. Linear probability model

**Fig. 6:**
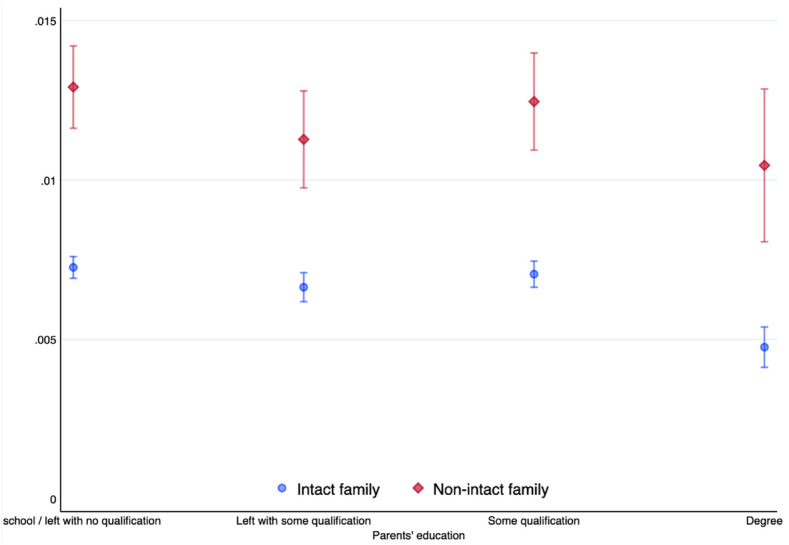
Predicted probability of offspring’s family dissolution by parents’ education. Discrete-time event history model

Figures 3 and 4 show that in all three cohorts, offspring from non-intact families were at greater risk of witnessing the break-up of their couples than offspring from intact families. The effect is smaller for the members of the Silent Generation, born between 1925 and 1945. Not only the separation rate of this generation was lower, but also the additional separation penalty of coming from a non-intact family was less sizeable. However, we have fewer observations for this oldest birth cohort, receive large standard errors and thus need to interpret results for this generation with caution (see Table 6 in the appendix for the regression table).

The main benefit of an analysis by birth cohort is to see how the parental class effect on family dissolution shifts over time. Among members of the Silent Generation, having parents with a higher socio-economic status was not associated with a lower risk of family dissolution. Only in the following cohorts of Baby Boomers and, above all, Generation X, was a more advantaged paternal class linked with a lower probability of seeing one’s own family break-up.

**Fig. 7:**
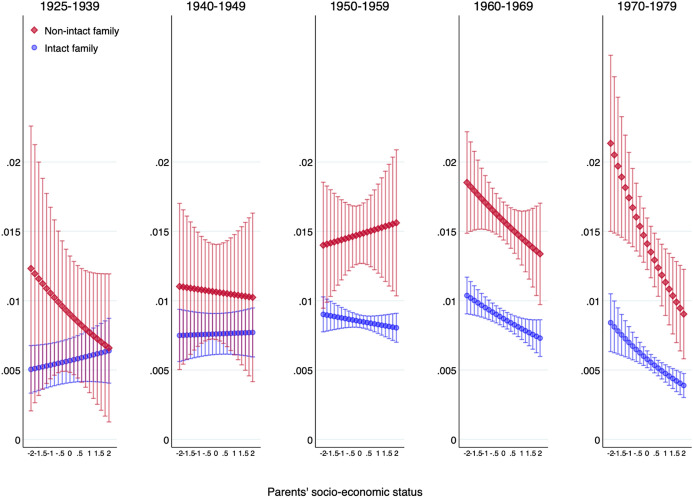
Predicted probability of offspring’s family dissolution by parents’ class for birth cohorts measured in decades. Discrete-time event history models

**Fig. 8:**
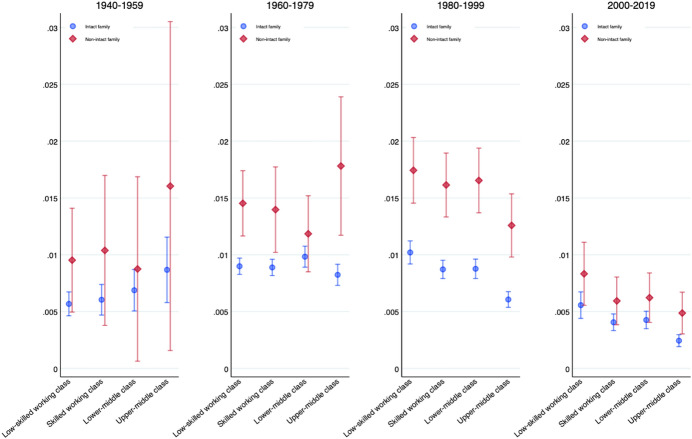
Predicted probability of offspring’s family dissolution by parents’ class for union cohorts. Discrete-time event history model

The reversal of the social-class gradient of union dissolution becomes even more evident if birth cohorts are measured by decades rather than sociological generations (see Fig. 7 in the appendix). However, family demographers often prefer to focus on union cohorts rather than birth cohorts. Figure 8 in the appendix shows that the conclusions of our analysis remain unchanged if we subdivide our analytical sample into four union cohorts. While an advantaged class background was associated with a higher risk of family dissolution in the oldest union cohort 1940–59, it became linked to a lower probability of family dissolution in the two youngest union cohorts of 1980–99 and 2000–19.

Contrary to our expectations, neither Figs. 3 and 4 on birth cohorts nor Fig. 7 on union cohorts point to a heterogeneous effect of parental class on the intergenerational transmission of separation. Socio-economic background seems to have a similar effect on offspring from intact and from non-intact families across cohorts, suggesting that there is no interaction effect between parents’ union dissolution and parents’ class position on the stability of children’s own childbearing union.

We turn again to a formal test and estimate the same model separately for each birth cohort. We avoid comparing odds ratios across different samples and show instead in Table 2 the coefficients of the linear probability model. In all three generations, growing up in a non-intact family is associated with greater instability in one’s own childbearing union. Contrary to Wolfinger’s (2011) result that the intergenerational transmission of divorce has weakened over time in the USA, we find no decreasing association of parents’ and offspring’s union dissolution for younger birth cohorts in the UK. The gap in the risk of union dissolution between children from intact and non-intact families increases from 9 percentage points in the Silent Generation to 13 points among Baby Boomers and members of Generation X.

**Table 2:** The effect of parents’ family status and social class on offspring’s family dissolution. Linear probability models

	Silent generation		Baby boomers		Generation X	
	1925–1945		1946–1964		1965–1979	
*Parents’ family status (ref: intact family)*						
Non-intact family	0.09**		0.14***		0.13***	
	(0.04)		(0.03)		(0.03)	
*Parents’ class (ref: low-skilled working class)*						
Skilled working class	0.01		− 0.02**		− 0.03**	
	(0.01)		(0.01)		(0.01)	
Lower-middle class	0.03*		− 0.01		− 0.04***	
	(0.01)		(0.01)		( − 0.01)	
Upper-middle class	0.01		− 0.06***		− 0.06***	
	(0.01)		(0.01)		( − 0.01)	
*Parents’ family status # parents’ class*						
Non-intact # skilled working class	0.03		− 0.03		0.00	
	(0.06)		(0.04)		(0.04)	
Non-intact # lower-middle class	− 0.06		− 0.01		− 0.03	
	(0.06)		(0.04)		(0.04)	
Non-intact # upper-middle class	− 0.02		0.04		− 0.01	
	(0.08)		(0.05)		(0.04)	
Observations (individuals)	7999		15,477		10,027	

Controls for year of birth, gender and ethnicity. Analytical samples only include individuals aged 40+

*p < .05

**p < .01

***p < .001

In terms of class origin, offspring from the low-skilled working class were no more likely to break-up their childbearing union in the Silent Generation, but most likely to do so in the two subsequent generations of Baby Boomers and, above all, Generation X. While these two main effects are large and statistically significant, there is no systematic interaction effect between parents’ family dissolution and parents’ social class for any of the three generations.

## Conclusion

Our study shows a powerful influence of parents’ family dissolution on children’s family dissolution, but it provides no evidence for a compensatory class effect that moderates this influence. Contrary to our expectation, offspring from more advantaged class backgrounds are not any less affected by their parents’ separation than offspring from less advantaged backgrounds. Therefore, coming from a privileged social origin does not weaken the link between parents’ family dissolution and offspring’s family dissolution in the UK.

At first glance, it may seem surprising that the influence of parental separation on offspring’s separation does not vary for children from different social origins. Although parents’ union dissolution tends to be an adverse life event for many children (but not for all, see Brand et al., 2019), the compensatory class effect does not set in for this type of adversity. Our study reported results both in relative terms using odds ratios and in absolute terms using predicted probabilities and coefficients from linear probability models (as suggested in Bernardi & Boertien, 2017b). Yet regardless of whether we focus on relative or absolute differences—or whether we use an ordinal class measure or a continuous socio-economic indicator –, we do not find any systematic heterogeneity in the effect that parents’ family status has on offspring’s family dissolution.

At second glance, the absence of a heterogeneous effect may be less surprising. The reason is that our analysis does not deal with the intergenerational transmission of socio-economic advantage, as in the literature on how parental divorce affects the education of children from different class origins. Rather, our analysis focuses on the transmission of behaviour—and parents’ socio-economic resources appear more consequential for children’s socio-economic attainment—be it education or occupation—than their behavioural outcomes such as partnership dissolution. For this later outcome, other mechanisms seem more central such as socialization in childhood—social learning and role models (Amato, 2000; Moen et al., 1997)—as well as the potential contribution of genetic inheritance (D’Onofrio et al., 2007; McGue & Lykken, 1992; Salvatore et al., 2018). In this sense, it may not come as a surprise that parents tend to transmit their partnership behaviour to their children to a similar extent, regardless of their position in the class hierarchy.

We further explored the possibility that the compensatory class effect is limited to the inheritance of union dissolution in younger birth cohorts. Although we observe a reversal of the social-class gradient in separation, the expectation of a heterogeneous effect for later cohorts was not borne out by the data either. Still, the reversal in the parental class gradient of family dissolution is of interest in and by itself, echoing the pattern shown for the link between individuals’ own education and union dissolution (Härkönen & Dronkers, 2006; Matysiak et al., 2014). In sociological research, large shifts in the association between socio-demographic characteristics are unusual. Even more unusual are reversals in the *direction* of relationships as the one observed between social origin and family dissolution over the birth cohorts of the 20th century. Our results thus confirm that marriage and the family are in a state of great flux in contemporary societies (Chan & Halpin, 2005; Cherlin, 2010).

Finally, we need to raise several caveats. All our findings only pertain to the couples of offspring who themselves had children—and not to childless couples. Moreover, our results of the intergenerational transmission of separation are likely an underestimation because we only account for respondents’ family status (whose parents had or had not separated) and ignore the family status of his or her partner. While we share this shortcoming with most studies in the field (see Gähler et al., 2009, p. 709), evidence for the USA suggests that couples in which both spouses experienced their parents’ divorce are more likely to divorce than couples in which only one spouse comes from a divorced family—and these couples are, in turn, more prone to divorce than couples where both partners come from intact families (Amato, 1996; Wolfinger, 2005). Therefore, comparing only couples where both partners come from separated families with couples where both partners come from intact families would likely produce even stronger evidence for the inheritance of union dissolution.

Finally, our results are limited to the UK—and it is unclear how well the British experience of union formation and dissolution generalizes to other countries. The UK could either be an *exception* or a *frontrunner*. When comparing eight European countries, Kalmijn and Leopold (2021) find the UK to be an *exception* in two regards. Its separation surge over the last decades was less stratified between the higher- and lower-educated, and it was less strongly concentrated among childless couples than elsewhere in Europe.

However, the UK may also be a *frontrunner* that has simply embarked earlier on a common European trajectory of family demography. This hypothesis is suggested by Brons and Härkönen (2018) who show that the association between parents’ education and children’s risk of union dissolution has become more negative across Europe—with the earliest and strongest reversal observed for the UK. Future studies will hopefully tell us how representative the UK’s shifting class pattern behind the inheritance of family dissolution is for other countries.

## Appendix

See Tables 3, 4, 5, 6, 7, 8.

**Table 3:** Descriptive statistics of the variables used

	*N*	*Mean*	*SD*
Analytical sample size	38,515		
Separation/divorce	6927	0.18	0.38
*Family status at age 16*			
Intact	31,959	0.16	0.37
Non-intact	3624	0.29	0.45
One or both parents died/other	2932	0.23	0.42
*Parents’ class*			
Low-skilled working class	9747	0.25	0.43
Skilled working class	9895	0.26	0.44
Lower-middle class	8324	0.22	0.41
Upper-middle class	7336	0.19	0.39
Missing	3213	0.08	0.28
Average ISEI of parents (Non standardized)		29.30	18.61
*Parents’ education*			
No schooling/no qualification	10,618	0.28	0.40
Left with some qualification	5670	0.15	0.35
Some qualification	6443	0.17	0.37
Degree	2414	0.06	0.24
*Gender*			
Male	18,079	0.47	0.50
Female	20,436	0.53	0.50
*Ethnicity*			
British/Irish	31,586	0.82	0.38
European/other white	1171	0.03	0.17
Mixed: white & other	400	0.01	0.10
Indian	1425	0.04	0.19
Pakistani	916	0.02	0.15
Bangladeshi	651	0.02	0.13
Other Asian/Asian British	599	0.02	0.12
Black/African/Caribbean/black British	1290	0.03	0.18
No information	115	0	0.05
*Cohort*			
Silent Generation: 1928–1945	7999	21.05	
Baby Boomers: 1946–1964	15,768	41.50	
Generation X: 1965–1979	14,224	37.44

**Table 4:** The effect of parents’ family status and parents’ social class on offspring’s family dissolution–linear probability model

	Coefficient	SE	Coefficient	SE
*Parents’ family status (ref: intact family)*				
Non-intact family	0.12***	(0.008)	0.13***	(0.017)
*Parents’ social class (ref: low-skilled working class)*				
Skilled working class	− 0.01**	(0.006)	− 0.01**	(0.006)
Lower-middle class	− 0.01	(0.006)	− 0.01	(0.007)
Upper-middle class	− 0.05***	(0.006)	− 0.05***	(0.007)
*Parents’ family status # parents’ social class*				
Non-intact # skilled working class			− 0.01	(0.025)
Non-intact # lower-middle class			− 0.04	(0.024)
Non-intact # upper-middle class			− 0.00	(0.027)
Observations (individuals)	34,027		34,027	

Controls are included for year of birth, gender and ethnicity. The analytical sample of the linear probability model only includes individuals aged 40 and older

**p* < .05

***p* < .01

****p* < .001

**Table 5:** The effect of parents’ family status and class on offspring’s family dissolution—controlling for socio-demographic and behavioural mediators. Linear probability model

	M1: no controls	M2: socio-demo	M3: life course	M4: all controls
*Parents’ family status (ref: intact Family)*				
Non-intact	0.13***	0.13***	0.10***	0.09***
	(0.02)	(0.02)	(0.02)	(0.02)
One or both parents died	0.06***	0.06***	0.04***	0.04***
	(0.02)	(0.02)	(0.02)	(0.02)
*Dominant class (ref: low-skilled working class)*				
Skilled working class	− 0.01**	− 0.01**	− 0.00	− 0.00
	(0.01)	(0.01)	(0.01)	(0.01)
Lower-middle class	− 0.01	− 0.01	0.02**	0.02***
	(0.01)	(0.01)	(0.01)	(0.01)
Upper-middle class	− 0.06***	− 0.05***	− 0.01*	− 0.00
	(0.01)	(0.01)	(0.01)	(0.01)
*Parents’ family status # parents’ class*				
Non-intact # skilled working class	− 0.02	− 0.01	− 0.01	− 0.01
	(0.02)	(0.02)	(0.02)	(0.02)
Non-intact # lower-middle class	− 0.04	− 0.04	− 0.03	− 0.03
	(0.02)	(0.02)	(0.02)	(0.02)
Non-intact # upper-middle class	0.01	− 0.00	− 0.00	− 0.00
	(0.02)	(0.02)	(0.02)	(0.02)
*Controls included:*				
Socio-demographics: gender, birth year, ethnicity		x	x	x
Union formation behaviour: age at union formation, married, previous unions, N° children			x	x
Own education				x
Observations	34,027	34,027	34,027	34,027
R-squared	0.01	0.03	0.12	0.12

Analytical samples only include individuals aged 40 and older

**p* < .05

***p* < .01

****p* < .001

**Table 6:** The effect of parents’ family status and class on offspring’s family dissolution–controlling for socio-demographic. By cohorts. Linear probability model

	Silent generation: 1925–1945		Baby Boomers: 1946–1964		Generation X: 1965–1979	
*Parents’ family status (ref: intact family)*						
Non-intact family	0.09***	(0.036)	0.14***	(0.027)	0.13***	(0.029)
Parents’ class (ref: low-skilled working class)	− 0.04*	(0.021)	− 0.05***	(0.017)	− 0.05***	(0.013)
Missing class	− 0.04*	(0.021)	− 0.05***	(0.017)	− 0.05***	(0.013)
Skilled working class	0.01	(0.012)	− 0.02**	(0.010)	− 0.03**	(0.012)
Lower-middle class	0.03**	(0.014)	− 0.01	(0.010)	− 0.04***	(0.012)
Upper-middle class	0.01	(0.015)	− 0.06***	(0.010)	− 0.06***	(0.011)
Parents’ family status # parents’ class						
Non-intact # missing class	0.04	(0.068)	− 0.00	(0.048)	0.08*	(0.047)
Non-intact # skilled working class	0.03	(0.063)	− 0.03	(0.040)	0.00	(0.039)
Non-intact # lower-middle class	− 0.06	(0.060)	− 0.01	(0.040)	− 0.03	(0.037)
Non-intact # upper-middle class	− 0.02	(0.078)	0.04	(0.047)	− 0.01	(0.039)
Gender (ref: male)						
Female	0.04***	(0.009)	0.05***	(0.007)	0.05***	(0.007)
Year of birth	0.00***	(0.001)	− 0.00	(0.001)	− 0.01***	(0.001)
Year of birth squared	0.00***	(0.001)	− 0.00	(0.001)	− 0.01***	(0.001)
*Ethnicity (ref: British/white)*						
No information	− 0.02	(0.108)	− 0.19***	(0.022)	− 0.11***	(0.021)
European/other white	− 0.07**	(0.028)	− 0.04*	(0.021)	− 0.04***	(0.016)
Mixed: white & other	0.17**	(0.087)	− 0.02	(0.035)	0.02	(0.030)
Indian	− 0.17***	(0.012)	− 0.18***	(0.009)	− 0.11***	(0.009)
Pakistani	− 0.12***	(0.032)	− 0.17***	(0.014)	− 0.10***	(0.011)
Bangladeshi	− 0.12***	(0.043)	− 0.18***	(0.015)	− 0.14***	(0.009)
Other Asian/Asian British	− 0.14***	(0.043)	− 0.12***	(0.021)	− 0.10***	(0.014)
Black/African/Caribbean/black British	0.05	(0.036)	0.00	(0.021)	− 0.03**	(0.016)
Other	0.02	(0.068)	− 0.04	(0.038)	− 0.10***	(0.021)
Constant	− 9.29***	(1.686)	0.88	(1.164)	14.65***	(1.589)
Observations	7,999		15,477		10,027	

Controls for year of birth, gender and ethnicity. Analytical samples only include individuals aged 40+

**p* < .05

***p* < .01

****p* < .001

**Table 7:** The effect of parents’ family status and class on offspring’s family dissolution—four model specifications. Discrete-time event history logit

	1. No controls	2. Socio-demo	3. Life course choices	4. All controls
*Family status at 16*				
Non-intact	2.086***	1.745***	1.806***	1.800***
	(0.070)	(0.058)	(0.084)	(0.084)
One or both parents died	1.336***	1.327***	1.343***	1.351***
	(0.064)	(0.064)	(0.085)	(0.086)
Normalized score of ISEI	1.034**	0.943***	0.991	1.028
	(0.015)	(0.015)	(0.020)	(0.022)
*Family status at 16 # ISEI*				
Non-intact # ISEI	0.928**	0.989	0.979	0.974
	(0.032)	(0.034)	(0.047)	(0.047)
One or both parents died # ISEI	0.993	1.020	1.027	1.022
	(0.050)	(0.052)	(0.067)	(0.067)
Time	1.895***	1.897***	1.986***	1.989***
	(0.034)	(0.034)	(0.038)	(0.038)
Time squared	0.962***	0.962***	0.961***	0.961***
	(0.001)	(0.001)	(0.001)	(0.001)
Time cubic	1.001***	1.001***	1.001***	1.001***
	(0.000)	(0.000)	(0.000)	(0.000)
Female		1.201***	0.907***	0.906***
		(0.028)	(0.028)	(0.029)
Year of birth		1.017***	1.015***	1.016***
		(0.001)	(0.002)	(0.002)
*Ethnicity*				
No information		0.440**	0.439*	0.416*
		(0.172)	(0.195)	(0.189)
European/other white		0.714***	0.722***	0.749***
		(0.060)	(0.076)	(0.079)
Mixed: white & other		1.140	1.398**	1.484***
		(0.123)	(0.206)	(0.221)
Indian		0.143***	0.111***	0.112***
		(0.022)	(0.019)	(0.019)
Pakistani		0.201***	0.145***	0.144***
		(0.031)	(0.026)	(0.026)
Bangladeshi		0.067***	0.042***	0.041***
		(0.021)	(0.014)	(0.014)
Other Asian/Asian British		0.311***	0.352***	0.371***
		(0.052)	(0.067)	(0.071)
Black/African/Caribbean/black British		1.004	1.293***	1.346***
		(0.067)	(0.112)	(0.117)
Other		0.661***	0.659**	0.690*
		(0.104)	(0.132)	(0.139)
Age of entry union w child			0.861***	0.864***
			(0.004)	(0.004)
*Number of unions with children*				
2nd union w child			0.701***	0.668***
			(0.063)	(0.060)
3rd union w child			0.456***	0.417***
			(0.102)	(0.094)
Ever in a marriage			1.002	0.992
			(0.088)	(0.088)
Married at the beginning of the union (direct marriage)			0.748***	0.740***
			(0.029)	(0.029)
*Education (Ref = Degree)*				
Other higher				1.063
				(0.060)
A level/similar				1.176***
				(0.057)
GCSE/similar				1.058
				(0.059)
Other qualification				0.897*
				(0.057)
No qualification/Missing				0.725***
				(0.043)
Constant	0.000***	0.000***	0.000***	0.000***
	(0.000)	(0.000)	(0.000)	(0.000)
Observations	958,240	958,240	958,240	958,240
Number of pidp	38,515	38,515	38,515	38,515

Robust standard errors in parentheses

^*^*p* < 0.1

^**^*p* < 0.05

^***^*p* < 0.01

**Table 8:** The effect of parents’ family status and class on offspring’s family dissolution—four model specifications. Linear probability model

	1. No controls	2. Socio-demo	3. Life course choices	4. All controls
*Family status at 16 *				
Non-intact	0.13***	0.12***	0.10***	0.10***
	(0.009)	(0.009)	(0.008)	(0.008)
One or both parents died	0.07***	0.07***	0.05***	0.05***
	(0.011)	(0.011)	(0.010)	(0.010)
Normalized score of ISEI	− 0.00	− 0.00	− 0.00	0.00
	(0.003)	(0.003)	(0.003)	(0.003)
Family status at 16 # ISEI	(0.000)	(0.000)	(0.000)	(0.000)
Non-intact # ISEI	− 0.02**	− 0.01	− 0.00	− 0.01
	(0.009)	(0.009)	(0.008)	(0.008)
One or both parents died # ISEI	− 0.01	− 0.01	0.00	0.00
	(0.011)	(0.011)	(0.010)	(0.010)
Time		0.05***	0.01***	0.01***
		(0.004)	(0.004)	(0.004)
Time squared		− 0.00***	− 0.00***	− 0.00***
		(0.000)	(0.000)	(0.000)
Time cubic		− 0.14***	− 0.07***	− 0.07***
		(0.018)	(0.021)	(0.021)
Female		− 0.06***	− 0.04***	− 0.04***
		(0.012)	(0.011)	(0.011)
Year of birth		0.01	0.04*	0.05**
		(0.022)	(0.021)	(0.021)
*Ethnicity*				
No information		− 0.16***	− 0.17***	− 0.16***
		(0.006)	(0.006)	(0.006)
European/other white		− 0.14***	− 0.16***	− 0.16***
		(0.008)	(0.009)	(0.009)
Mixed: white & other		− 0.16***	− 0.20***	− 0.20***
		(0.007)	(0.008)	(0.008)
Indian		− 0.12***	− 0.10***	− 0.09***
		(0.012)	(0.012)	(0.012)
Pakistani		− 0.01	0.02*	0.03**
		(0.012)	(0.012)	(0.012)
Bangladeshi		− 0.07***	− 0.06***	− 0.05**
		(0.020)	(0.019)	(0.020)
Other Asian/Asian British				0.04***
				(0.007)
Black/African/Caribbean/black British				0.06***
				(0.007)
Other				0.07***
				(0.007)
Age of entry union w child			− 0.01***	− 0.01***
			(0.000)	(0.000)
*Number of unions with children*				
2nd union w child				
3rd union w child				
Ever in a marriage			0.03***	0.02***
			(0.007)	(0.008)
*Education (Ref=Degree)*				
Other higher				0.04***
				(0.007)
A level/similar				0.06***
				(0.007)
GCSE/similar				0.07***
				(0.007)
Other qualification				0.07***
				(0.008)
No qualification/Missing				0.05***
				(0.006)
Constant	0.17***	1.61***	1.81***	1.38***
	(0.002)	(0.312)	(0.306)	(0.322)
Observations	34,027	34,027	34,027	34,027

Robust standard errors in parentheses

**p* < 0.1

***p* < 0.05

****p* < 0.01

See Figs. 5, 6, 7, 8.

## Footnotes

1 We measure parental education by taking the higher attainment of either father and mother and distinguish five levels: no school/no qualification (25.4%), dropout with some qualification (14.7%), some qualification (16.2%), degree (6.7%), missing information on education (37%). The large number of missing observation of parental education is largely random because this question was only asked in interviews held between January and June 2009 and not the rest of UKHLS wave 1 in order to reduce interview length.

2 We code occupations into classes based on the Standard occupational classification 2010 (SOC-2010 at the 3 digit-level) or 1990 (SOC-90 at the 2 digit-level). All the Stata codes are readily available from the authors.
